# Syndecan-4 promotes vascular beds formation in tissue engineered liver via thrombospondin 1

**DOI:** 10.1080/21655979.2020.1846897

**Published:** 2020-11-29

**Authors:** Xiaoyi Hu, Junjie Chen, Hechen Huang, Shengyong Yin, Shusen Zheng, Lin Zhou

**Affiliations:** a Division of Hepatobiliary and Pancreatic Surgery, Department of Surgery, The First Affiliated Hospital, Zhejiang University School of Medicine, Hangzhou, Zhejiang, China; b NHC Key Laboratory of Combined Multi-Organ Transplantation, Hangzhou, Hangzhou, China; c Key Laboratory of the Diagnosis and Treatment of Organ Transplantation, Research Unit of Collaborative Diagnosis and Treatment for Hepatobiliary and Pancreatic Cancer, Chinese Academy of Medical Sciences (2019RU019), Hangzhou, Zhejiang, China; d Key Laboratory of Organ Transplantation, Research Center for Diagnosis and Treatment of Hepatobiliary Diseases, Hangzhou, Zhejiang, China

**Keywords:** Syndecan-4, vascular bed formation, thrombospondin-1, tissue engineered liver

## Abstract

Instantaneous blood coagulation after bioengineered liver transplantation is a major issue, and the key process in its prevention is the construction of the endothelial vascular bed on biomimetic scaffolds. However, the specific molecules involved in the regulation of the vascular bed formation remain unclear. Syndecan-4 is a type I transmembrane glycoprotein commonly expressed in the human body; its receptor has been reported as critical for optimal cell adhesion and initiation of intracellular signaling, indicating its promising application in vascular bed formation. In the current study, bioinformatics analysis and in vitro experiments were performed to evaluate whether syndecan-4 promoted endothelial cell migration and functional activation. Exogenous syndecan-4-overexpressing endothelial cells were perfused into the decellularized liver scaffold, which was assessed by Masson’s trichrome staining. Western blotting and qRT-PCR were used to evaluate the effects of syndecan-4 on the thrombospondin 1 (THBS1) stability. We found that syndecan-4 promoted the adhesion of vascular endothelial cells and facilitated cell migration and angiogenesis. Furthermore, syndecan-4 overexpression resulted in a well-aligned endothelium on the decellularized liver scaffolds. Mechanistically, syndecan-4 destabilized THBS1 at the protein level. Therefore, our data revealed that syndecan-4 promoted the biological activity of endothelial cells on the bionic liver vascular bed through THBS1. These findings provide scientific evidences for solving transient blood coagulation after bionic liver transplantation.

## Graphical abstract

**Figure:**
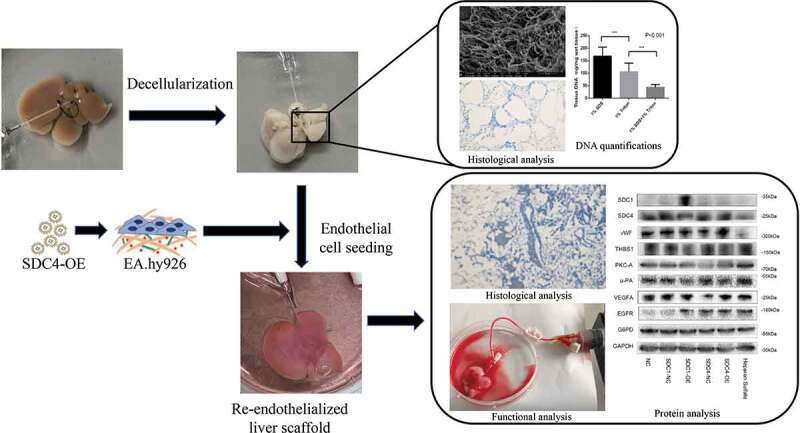

## 1. Introduction

End-stage liver disease (ESLD) is one of the most severe health problems worldwide and is characterized by high morbidity and mortality [1]. Until now, liver transplantation is considered the gold standard method for treating ESLD [2]. It has been reported that there are 15,000 patients awaiting liver transplantation, and only 6,000 liver transplants are performed yearly in the United States [3–5]. This disparity in organ supply and demand has created a problem worldwide that limits the widespread use of liver transplantation each year. Over the past decades, the development of regenerative medicine and tissue engineering technology points in a new direction for resolving the above-mentioned issues [6]. Tissue engineering is dedicated to providing an inexhaustible supply of transplantable tissues or whole organs for functional restoration. In particular, producing biomimetic tissue-engineered liver for transplantation into patients with ESLD will have a huge impact on the medical field and provide significant social benefits. Decellularization is a process wherein chemical or physical methods are used to remove cellular components of living tissues to obtain acellular extracellular matrix (ECM), which is considered the preferred method for application in tissue engineering and regenerative medicine [7–9]. Recently, bioengineered tissues with simple structures have been successfully constructed, including the liver, blood vessels, heart, and kidney [10–13]. Previously we showed that decellularization allows for efficient recellularization of the decellularized liver lobe matrix with allogenic primary hepatocytes in vivo [14]. However, a major obstruction in the transformation and clinical application of this liver bioengineering technology is the absence of vascular bed reconstruction to prevent transient coagulation after bionic liver organ transplantation.

Revascularization remains a major challenge for complex solid engineered organs. All tissue-engineered organs require a vascular network that supplies oxygen and nutrients, and its vascular structure can be directly connected to the circulatory system of the recipient. As the center of human metabolism, practical oxygen consumption of the liver cells requires that the liver mass is large enough to facilitate metabolic functions, such as a broad microvascular network connected to the blood supply; otherwise, the liver parenchymal cells will be damaged by ischemia [15]. Although decellularized scaffolds are characterized by the presence of a complete vascular network, the interaction of the ECM collagen with various components in the blood leads to the transient coagulation cascade due to the lacking endothelial cell lining in decellularized liver scaffolds. Thrombosis is a common complication arising after subsequent in vitro blood perfusion or in vivo bionic liver transplantation [16–19]. Currently, microvascular endothelial cells, human umbilical vein endothelial cells, and endothelial progenitor cells have been reported in re-endothelialization of decellularized liver scaffolds to prevent blood clots [20]. However, making the endothelial cells well aligned on the surface of the vascular catheter remains a challenge.

**Figure 1.**
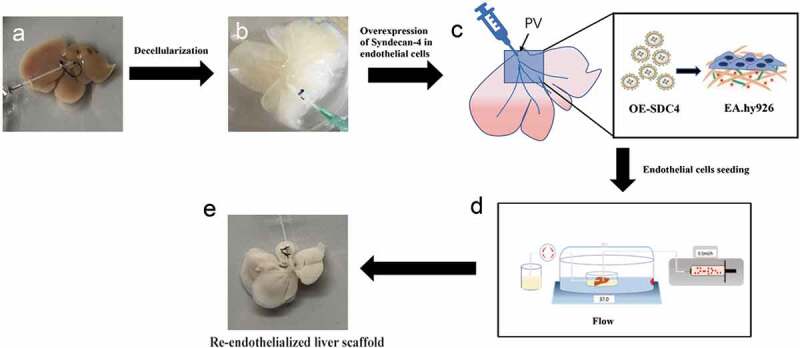
Schematic diagram of the re-endothelialization processes for decellularized liver scaffolds

The aim of the current study was to explore a potential endogenous factor for the promotion of re-endothelialized liver scaffolds. In our study, we developed a novel re-endothelialization technique using bioinformatics tools and evaluated the role of syndecan-4 in the formation of vascular beds. We validated its functionality by re-endothelializing mice livers using microvascular endothelial cells overexpressing syndecan-4 (Figure 1). Therefore, the major findings of the study might provide a new scientific basis for the reconstruction of blood flow post biomimetic liver transplantation.

(a). Native liver harvested from mice was decellularized using detergent solution to obtain a completely decellularized liver scaffold (b). Overexpression of syndecan-4 in endothelial cells (c), the scaffold was seeded with SDC-4 overexpressing endothelial cells using a combination of static (d), and perfusion methods and then matured in a bioreactor system. (e). The engineered liver was constructed. PV indicates portal vein.

## 2. Materials and methods

### 2.1. Decellularization of the rat liver

Native livers were harvested from C57BL/6 mice weighing 70–80 g after peritoneal anesthesia with chloral hydrate. The portal vein (PV) was cannulated with smart site connectors (Cole Parmer, USA) attached to 14 G tubing with the inflow and outflow adjusted to mimic normal flow through the organ. Detergent solutions (1% sodium dodecyl sulfate in saline for 1 h, 1% Triton X-100 for 1 h) were perfused into the liver tissue using a peristaltic pump (Master flex, USA). Decellularization of the liver was performed by perfusing the organ at a flow rate of 0.5 ml/min for 48 h, followed by washing with saline for 12 h. All the perfusion experiments were performed in a bioreactor system at 37°C.

### 2.2. Characterization of the acellular liver scaffold

To evaluate the efficiency of scaffold decellularization, DNA quantification, scanning electron microscopy (SEM), and Masson’s trichrome staining were performed. For DNA quantification, samples were excised from the representative lobes of native and decellularized livers. The samples were minced and lyophilized for quantitative analysis. DNA was extracted from 5 mg samples using the Tissue Kit (Qiagen, GER) and quantified using the Quant-iT PicoGreen (Invitrogen, USA). For Masson’s trichrome staining, the sections were stained using the Masson’s Trichrome Stain kit (Sigma-Aldrich, GER, CS0760-1KT) according to the manufacturer’s instructions.

### 2.3. Cell culture and lentiviral infection

Two human endothelial cells lines, EA.hy926 and HMEC-1, were obtained from the Cell Bank of the Shanghai Institutes of Biological Sciences, Chinese Academy of Sciences. EA.hy926 and HMEC-1 were cultured in RPMI 1640 medium (Biological Industries, IL) with 10% fetal bovine serum (FBS, Biological Industries, IL) at 37°C in a 5% CO2 incubator (Thermo Scientific, USA). For syndecan-4 upregulation, lentivirus was purchased from GeneChem (China). The two endothelial cells lines were used for the overexpression of syndecan-4 using a lentivirus at a multiplicity of infection (MOI) of 30 pfu per cell, according to the manufacturer’s instructions. Stably transfected cells were selected using 4 μg/ml puromycin (Sigma-Aldrich, USA) for 3 days.

### 2.4. Wound healing and adhesion assays

The capability of HMEC-1 cells to migrate was evaluated by a scratch motility assay. HMEC-1 cells (4 × 10^6^ cells per well) were seeded in a 6-well plate and grown overnight to achieve confluence in the RPMI 1640 medium supplemented with 10% FBS. Next, the cells were serum-starved for 24 h in the RPMI 1640 medium (2% FBS). The monolayer was scratched with a pipette tip, washed with phosphate-buffered saline (PBS) to remove floating cells, and then cultured in RPMI 1640 medium with 10% FBS for 18 h. The scratched area was then photographed, and the distance of wound healing in each well was evaluated. Five hundred cells were loaded onto 96-well cell culture plates coated with fibronectin (FN), collagen I and IV, and laminin and allowed to adhere for 1 h before assessing cell adhesion. Adherent cells were stained and counted using a microscope by an investigator blinded to the experimental set-up.

### 2.5. In vitro angiogenesis assay

Matrigel liquefaction (Becton, Dickinson and Company, USA) was performed under 4°C for 12 h. Each well of a 96-well cell culture plate was supplemented with 50 μl Matrigel, and the plate was placed in an incubator for 60 min to solidify the Matrigel. HMEC-1 cells were seeded at a concentration of 5 × 10^4^ cells per well in the plate and cultivated in an incubator for 12 h. Next, the formation of tubular structures was observed under an inverted microscope.

### 2.6. Re-endothelialization of decellularized liver scaffolds and coagulation assay

To improve the re-endothelialization of vasculatures within the liver scaffold, human vascular endothelial cells overexpressing syndecan-4 were used. Cannula in the portal vein and hepatic artery of the decellularized liver stent was used as double inlet and the vena cava was used as the outlet to establish a closed circulation perfusion device consisting of a peristaltic pump and a heater (retain 37°C). The RPMI 1640 medium (with 10% FBS) was circulated and perfused through the right branches of the portal vein and hepatic artery at the flow rates of 2 ml/min and 0.4 ml/min, respectively. After 30 min of culture medium infusion, 1 × 10^6^ EA.hy926 cells in total were perfused into the portal vein and hepatic artery four times, each time for 15–20 min, to allow the endothelial cells to adhere to the ECM completely. For coagulation assay, peripheral blood voluntarily collected from the first author was diluted with two times a volume of 0.9% physiological saline with heparin (200 UI/ml). The mixture was then perfused into the re-endothelialized liver using the perfusion device at 2 ml/min to obverse the blood coagulation and clotting formation.

### 2.7. RNA extraction and qRT-PCR

RNA was extracted from the cells using the TRIzol reagent (Invitrogen, USA). A SYBR Premix ExTaq system (Takara, China, RR001A) and a Bio-Rad PCR instrument (USA) were used to perform the qRT-PCR.

### 2.8. Western blotting

Cells were lysed using the RIPA buffer. The BCA method was used to detect the thrombospondin-1 (THBS1) protein concentration. Next, 25 μg of protein was loaded onto 4–20% Express PLUSTMPAGE gels (GenScript, USA) for separation, followed by transfer onto polyvinylidene difluoride (PVDF) membranes. The membranes were incubated with a primary antibody overnight. Subsequently, the membranes were incubated with an HRP-conjugated secondary antibody (1:5000). The proteins were detected using the EZ-ECL kit (Biological Industries, Israel). The following antibodies were obtained from Abcam (Cambridge, UK): anti-SDC1 (ab128936), anti-SDC4 (ab74139), anti-vWF (ab134193), anti-THBS1 (ab1823), and anti-PKC-A (ab32326), anti-uPA (ab3218106), anti-VEGFA (ab1316), anti-EGFR (ab52894), anti-G6PD (ab210702), anti-GAPDH (ab8245).

### 2.9. Statistical analysis

Data were expressed as mean ± standard deviation. The statistical analysis was performed using SPSS 22.0 software (IBM Corp., Armonk, NY, USA). Student’s t-test was used to identify significant differences. One-way analysis of variance (ANOVA) was also used to analyze the data of cell migration and angiogenesis, the third group served as a reference for pairwise comparisons. A P-value <0.05 was considered statistically significant. *represented P < 0.05; **represented P < 0.01; and ***represented P < 0.001.

## 3. Results

### 3.1. Characterization of decellularized liver scaffolds

**Figure 2.**
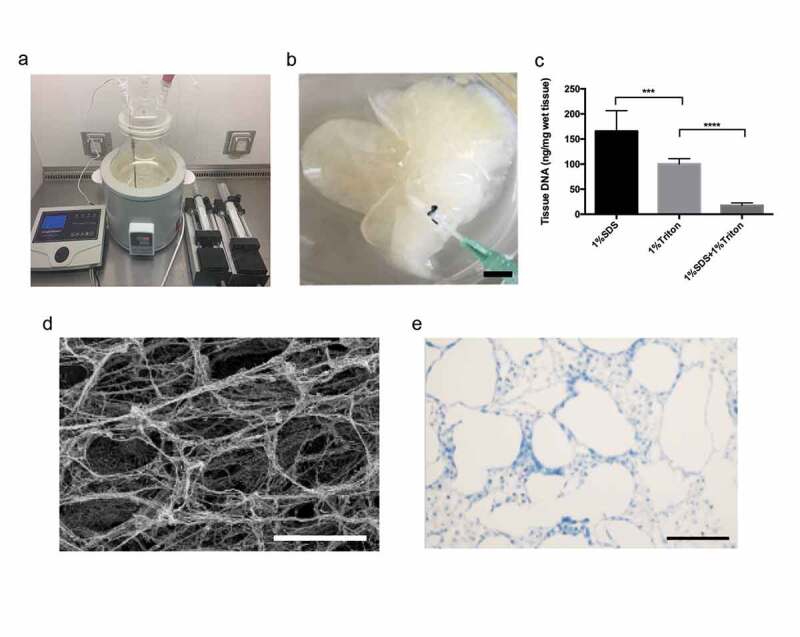
Decellularization of the liver scaffolds

A completely decellularized liver scaffold was obtained with the perfusion of detergent solutions through the portal vein (PV) using a peristaltic pump in a heating system (keep the chamber at a constant temperature of 37°C) (Figure 2(a)). Apart from gradually changing the color to white (Figure 2(b)), indicating that the cellular components were removed completely from the native tissue, the decellularized liver scaffolds maintained the volume and shape of the native liver. Residual DNA quantifications of scaffolds revealed a remarkable reduction in the amounts of DNA with progressing decellularization (Figure 2(c)). To examine the retention of intact blood vessels within the acellular liver scaffolds, SEM and Masson’s trichrome staining were performed. SEM analysis of the ECM of liver scaffolds showed a mesh-like appearance without any cellular components (Figure 2(d)). Importantly, Masson’s trichrome staining (Figure 2(e)) confirmed the presence of a fine scaffold structure within the parenchymal lobule, even after removing all the cells.

(a). A combination of peristaltic pump and heating system with bioreactor system (b). Gross image of mice liver decellularization (c). The quantification of residual DNA showed a significant reduction in DNA during the decellularized progress (d). SEM image of decellularized liver showing the honeycomb structure of extracellular matrix (e). Massons trichrome staining showing the sinusoid-like structures of decellularized liver. Scale bars: 5 mm (b), 20 μm (d), and 50 μm (e).

### 3.2. Identification of the re-endothelialization-associated hub gene

**Figure 3.**
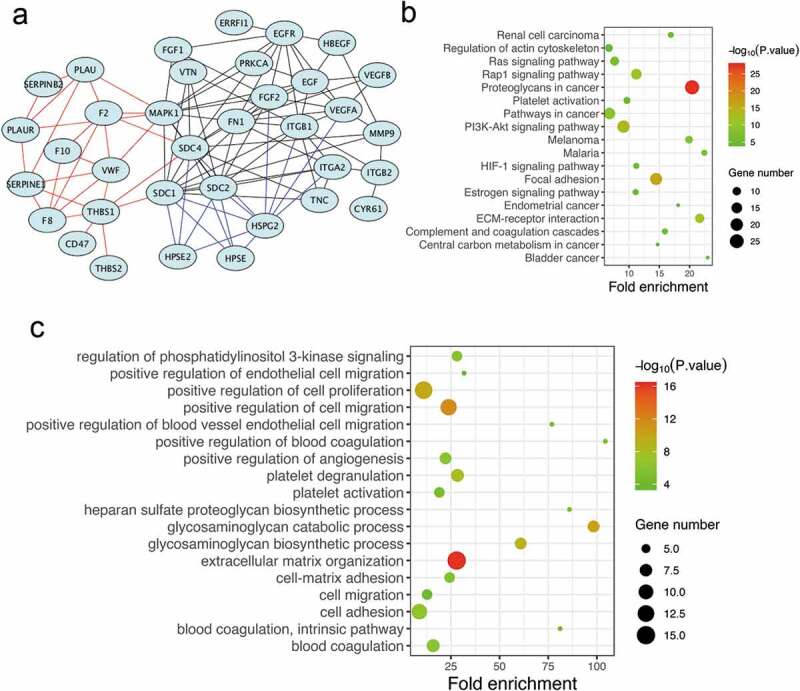
Functional enrichment and PPI network analysis of re-endothelialization-associated genes

Endogenously expressed and functional re-endothelialization factors are critical for maintaining vascular patency in the bionic liver. We aimed to identify the potential co-expressed genes of the syndecan family by analyzing the differential genes from the two data sets (GSE37843 & GSE21401). The protein–protein interaction (PPI) network was constructed by the STRING database (Version 11.0), and the syndecan family genes, especially syndecan-4, were selected as the hub gene (Figure 3(a)). To confirm the potential mechanism, we further performed pathway enrichment analyses using DAVID (Version 6.8). It showed that the syndecan protein family was mainly involved in focal adhesion, regulation of actin cytoskeleton, complement and coagulation cascades, and platelet activation (Figure 3(b)). The main pathways associated were organization of the ECM, positive regulation of cell proliferation, positive regulation of cell migration, and positive regulation of angiogenesis (Figure 3(c)). Therefore, our findings suggested that the syndecan family-associated genes, especially syndecan-4, might be the hub genes involved in endothelial cell adhesion, proliferation, and migration and anticoagulation/antithrombotic processes, which play important roles in re-endothelialization.

(a). PPI network was constructed by the CytoHubba. Functional and pathway enrichment analysis of related genes (b) GO analysis (c) KEGG analysisBP: Biological process; KEGG: Kyoto Encyclopedia of Genes and Genome.

### 3.3. Effects of syndecan-4 on re-endothelialization ability of vascular endothelial cells

**Figure 4.**
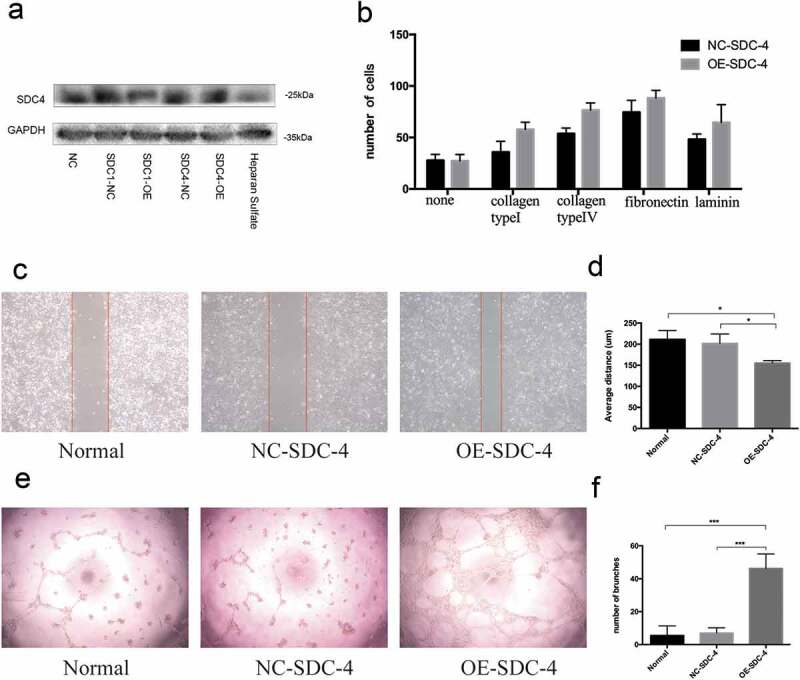
Effect of SDC-4 on ECs attachment and migration

To investigate whether overexpressing syndecan-4 could promote the biological effects of re-endothelialization, EA.hy926 cells were transfected with lentivirus harboring syndecan-4. The expression of syndecan-4 protein was significantly upregulated upon transfection (Figure 4(a)). The wound healing, cell adhesion, and Matrigel tube formation assays were performed to validate the angiogenic effect of syndecan-4 on vascular endothelial cells. Interestingly, we found that EA.hy926 cells overexpressing syndecan-4 displayed a higher level of cell adhesion compared with the control group (Figure 4(b)). Furthermore, cellular motility, or wound healing, was significantly enhanced in syndecan-4-overexpressing cells within 18 h of scratching (Figure 4(c,d)). Moreover, overexpression of syndecan-4 induced greater tube-like structure formation in the Matrigel compared with the control group (Figure 4(e,f)), suggesting that syndecan-4 promoted EA.hy926 angiogenesis in vitro. These results indicated that overexpression of syndecan-4 could induce the migration and adhesion of endothelial cells upon re-endothelialization.

(a). The efficiency of SDC-4 overexpressing in HMEC-1 cells (b). Real-time adhesion of endothelial cells on collagen type V, fibronectin, or laminin over 1 h (c). For wound healing assay, cell migration was recorded 18 hours after scratching. (d). Quantification of scratch widths. (e,f). The angiogenesis assay and the counting of closed tube-like structures per visual field (n =  3) revealed that SDC-4-overexpressing cells form better tube-like structures on Matrigel compared with the vector-transfected control group.

### 3.4. Re-endothelialization of the decellularized liver scaffold

**Figure 5.**
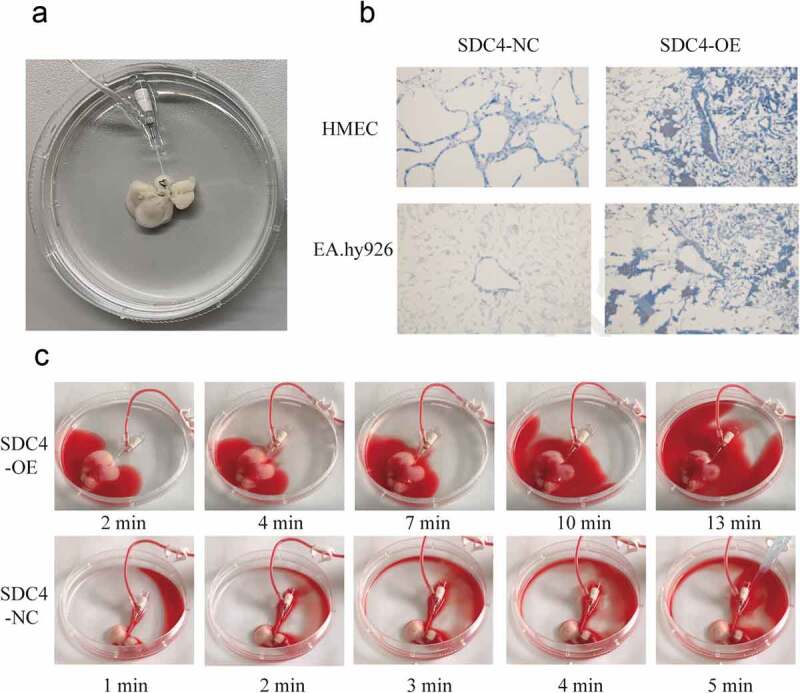
Re-endothelialization of liver scaffolds using syndecan-4 overexpressing EA.hy926

We next confirmed the effect of syndecan-4 on re-endothelialization of the acellular liver scaffolds by seeding EA.hy926 cells onto the scaffold using a combination of static and perfusion techniques (Figure 5(a)). Masson’s trichrome staining demonstrated that EA.hy926 cells overexpressing syndecan-4 were intact, well aligned, and uniformly distributed over the lumen of the large vessels (Figure 5(b)). These findings indicated that the overexpression of syndecan-4 in endothelial cells resulted in the formation of a uniform endothelial layer. Next, we focused on the coagulation of re-endothelialized liver scaffold. Heparinized blood diluted in saline solution (1:2) was pumped into repopulated liver. A total of 25 ml perfusion was distributed along the vascular bed from the PV to the liver periphery. The whole perfusion progress in re-endothelialized liver scaffold with syndecan-4-overexpressing EA.hy926 cells lasted more than 13 minutes without clotting (Figure 5(c)). However, the perfusion in syndecan-4-control re-endothelialized liver scaffold lasted only 4–5 minutes and ended with blood coagulation. We also measured the flow rates in the repopulated liver, the flow rates were about 1–1.3 ml/min (Supplementary Material).

(a). A reendothelialized liver scaffolds was constructed. (b). Representative images of liver scaffolds sections by Massons Trichrome staining. (c). The perfusion of diluted blood in re-endothelialized liver scaffold. Upper: re-endothelialized liver scaffold with syndecan-4-overexpressing EA.hy926 cells. Bottom: re-endothelialized liver scaffold with syndecan-4-NC EA.hy926 cells. Scale bars: 1 cm (a), 50 μm (b).

### 3.5. Syndecan-4 regulated re-endothelialization via THBS1

**Figure 6.**
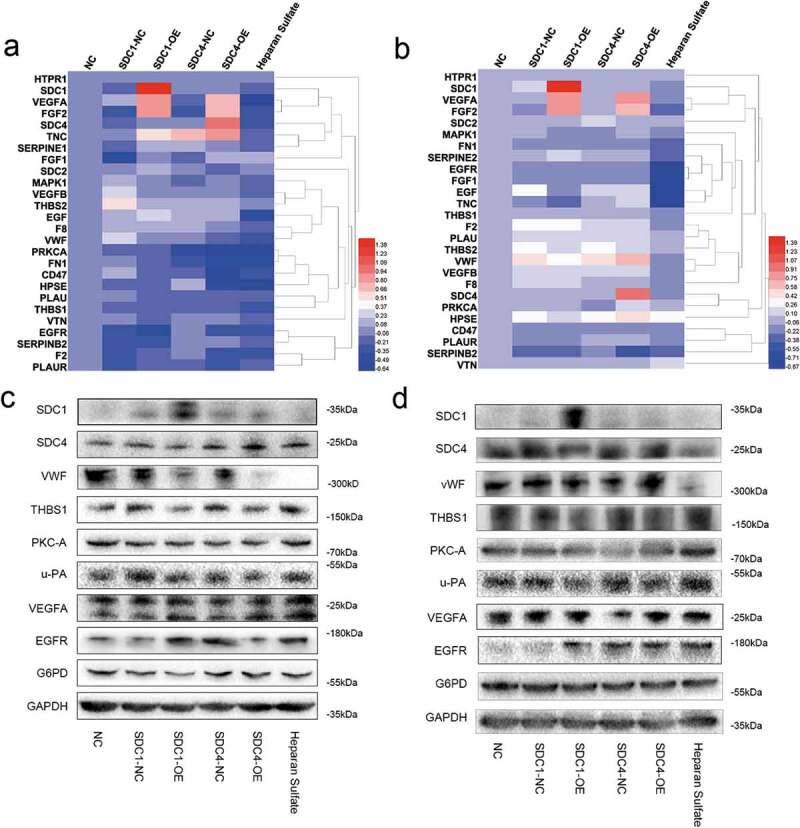
Syndecan-4 promotes re-endothelialization through THBS1

As shown in Figure 4(b), there were dramatic differences in adherence to collagen type V, fibronectin, or laminin between syndecan-4-overexpressing and control cells. Therefore, we speculated that syndecan-4 might regulate the adhesion of endothelial cells through THBS1 owing to its interaction with the matrix metalloproteinase, fibrinogen, fibronectin, laminin, collagen type V, and integrin α-V/β-1. To investigate the mechanism underlying the progression of re-endothelialization, qRT-PCR and western blot analysis were performed to evaluate the expression of THBS1 and other endothelial cell adhesion- and migration-related vascular factors after overexpression of syndecan-4. We found that the overexpression of syndecan-4 decreased the expression level of THBS1 protein, while the mRNA level of *THBS1* remained unchanged (Figure 6(a–d)). Another member of the syndecan family, syndecan-1 had no significant effect on the regulation of THBS1. These results verified that syndecan-4 downregulated the expression of THBS1 to regulate re-endothelialization in the bionic liver scaffolds.

(a,b). mRNA levels of THBS1 and other endothelial cell adhesion and migration-related vascular factors by qRT-PCR in SDC-4 and SDC-1 overexpression cells (c,d). Protein levels of THBS1 and other endothelial cell adhesion and migration-related vascular factors by western blot in SDC-4 and SDC-1 overexpression cells.

## 4. Discussion

Previous studies have shown that tissue-engineered livers are an attractive organ replacement solution for compensating the shortage of liver sources. Transplanting cells into a liver matrix made up of a decellularized scaffold that mimics the microstructure of native tissues is expected to provide a functional bionic liver [21–27]. Our study demonstrated that detergent perfusion through the portal vein created a translucent liver matrix while maintaining the native vasculature for adequate perfusion of constructs that were able to withstand physiological blood pressures. We also confirmed that the scaffold was devoid of DNA and preserved the three-dimensional architecture, indicating that our decellularization technique was efficient. Although it has been reported that sodium dodecyl sulfate is better able than Triton X to decellularize organs, in terms of ECM retention and DNA removal [28,29], Triton X is better at maintaining the micro-architecture than sodium dodecyl sulfate [30]. Consistently, our study showed the great ability of the combination of sodium dodecyl sulfate and Triton X in removing the pathogenic and antigenic epitopes, in addition to the retention of the ECM contents.

Previously, we have shown that transient coagulation after biomimetic tissue-engineered liver transplantation appears to be a bottleneck in the development of decellularized liver tissue engineering [16], and the key to solving this problem is the re-endothelialization of the scaffold. It is necessary to re-endothelialize the tissue-engineered liver completely within a reasonable period, in order to allow oxygen and nutrients to diffuse to the center of the bionic liver. Although innumerable studies have met with success in re-endothelialization, equivalent level of success has not been achieved in preventing thrombosis and delivering blood and nutrients to the liver. The Hussein group reported that a heparin-gelatin mixture could be used as an anticoagulant coating for inducing the adhesion and migration of endothelial cells on the surface of the tissue-engineered liver vascular beds [31]. The porcine liver acellular scaffold constructed by the Wu group promotes the connection of VEGF and heparinized scaffolds before endothelial cells are implanted via end-point attachment of heparin [32]. Accordingly, identifying endogenously expressed vasoactive factors that act locally in endothelial cells to enhance re-endothelialization efficacy is of great importance to prevent thrombosis. In our study, syndecan-4 was found to have the potential to regulate the formation of vascular beds on decellularized liver matrices. Syndecan-4 is a type I transmembrane glycoprotein that is commonly expressed in the human body [33]. Owing to its ubiquitous nature, syndecan-4 has an extracellular domain that binds to many components of the cellular microenvironment, such as cytokines or ECM [34]. Recent studies suggested that the syndecan-4 receptor is critical for optimal cell adhesion and initiation of intracellular signaling, thereby indicative of its promising application in the formation of vascular beds [35,36]. It has been reported that knockdown of syndecan-4 in endothelial cells leads to poor organization of endothelial cells under flowing conditions both in vitro and in vivo [37]. In our study, we found that syndecan-4 overexpression increased cell migration without affecting the viability of cells compared with the control group, and all endothelial cells were able to successfully proliferate, thus forming the tubular structure. In addition, overexpressing syndecan-4 also increased cell attachment. The density of syndecan-4-overexpressing endothelial cell adhesion was found to be high, especially with the fibrinogen, collagen type V, and laminin coating. We further demonstrated that THBS1 showed a high affinity for syndecan-4. It is suggested that syndecan-4 might competitively inhibit THBS1 anchoring to the cell membrane through physical binding, regulate protein translocation, localization, and chemical modification, increase the soluble components of THBS1, and weaken the ability of THBS1 to inhibit angiogenesis [38]. This might help explain the increase in cellular attachment of syndecan-4-overexpressing endothelial cells by interaction between fibronectin, laminin, and THBS1. Therefore, we believe that syndecan-4 is a key molecule regulating endothelial cell function and participates in vascular bed formation. However, several studies have reported that high expression of syndecan-4 is likely involved in carcinogenesis and cancer progression [39–41], indicating that overexpression of syndecan-4 may lead endothelial cells to rapidly proliferate and exhibit malignant features. Hence, further studies are warranted to identify a different mode of control of syndecan-4 expression to help safely establish a tissue-engineered liver with a fine scaffold structure.

In conclusion, we have demonstrated for the first time that transmembrane glycoprotein syndecan-4 could effectively promote endothelial cell colonization and adhesion and form a tube into a bionic scaffold, which would be feasible to generate bionic liver scaffolds mimicking native liver ECM with an intact vascular network extending to the capillary bed. Furthermore, we unveiled the potential regulation of THBS1 by syndecan-4, wherein syndecan-4 promoted the biological activity of endothelial cells through destabilizing THBS1 in the engineered liver vascular bed. Therefore, our findings could provide a scientific basis for solving the existing issue of transient coagulation after bionic liver transplantation, and thus improve its applicability.

## Supplementary Material

Supplemental Material

## Research highlights

- Syndecan-4 promoted endothelial cell adhesion and enabled re-endothelialization of decellularized liver scaffolds.
- Syndecan-4 promoted the formation of well-aligned endothelium structures.
- Syndecan-4 destabilized thrombospondin 1 to enable angiogenesis.

## References

[1] Sarin SK, Choudhury A. Acute-on-chronic liver failure: terminology, mechanisms and management. Nat Rev Gastroenterol Hepatol. 2016;13:131–149. 26837712 10.1038/nrgastro.2015.219

[2] Wang Y, Nicolas CT, Chen HS, et al. Recent advances in decellularization and recellularization for tissue-engineered liver grafts. Cells Tissues Organs. 2017;203:203–214. 28030865 10.1159/000452761

[3] Bernal W, Auzinger G, Dhawan A, et al. Acute liver failure. Lancet. 2010;376:190–201. 20638564 10.1016/S0140-6736(10)60274-7

[4] Boulter L, Lu WY, Forbes SJ. Differentiation of progenitors in the liver: a matter of local choice. J Clin Invest. 2013;123:1867–1873. 23635784 10.1172/JCI66026 PMC3635730

[5] Briceno J, Padillo J, Rufián S, et al. Assignment of steatotic livers by the mayo model for end-stage liver disease. Transpl Int. 2005;18:577–583. 15819807 10.1111/j.1432-2277.2005.00091.x

[6] Atala A. Organ preservation, organ and cell transplantation, tissue engineer- ing, and regenerative medicine: the terms may change, but the goals remain the same. Tissue Eng Part A. 2014;20:445e6. 24367926 10.1089/ten.TEA.2013.0742

[7] Gupta SK, Mishra NC, Dhasmana A. Decellularization methods for scaffold fabrication. Methods Mol Biol. 2018;1577:1–10. 28550502 10.1007/7651_2017_34

[8] Badylak SF, Freytes DO, Gilbert TW. Extracellular matrix as a biological scaffold material: structure and function. Acta Biomater. 2009;5:1–13. 18938117 10.1016/j.actbio.2008.09.013

[9] Gilbert TW, Sellaro TL, Badylak SF. Decellularization of tissues and organs. Biomaterials. 2006;27:3675–3683. 16519932 10.1016/j.biomaterials.2006.02.014

[10] Hoerstrup SP, Cummings Mrcs I, Lachat M, et al. Functional growth in tissue- engineered living, vascular grafts: follow-up at 100 weeks in a large animal model. Circulation. 2006;114:I159–66. 16820566 10.1161/CIRCULATIONAHA.105.001172

[11] Hussein KH, Park KM, Kim HM, et al. Construction of a biocompatible decellularized porcine hepatic lobe for liver bioengineering. Int J Artif Organs. 2015;38:96–104. 25744195 10.5301/ijao.5000394

[12] Ott HC, Matthiesen TS, Goh SK, et al. Perfusion- decellularized matrix: using nature’s platform to engineer a bioartificial heart. Nat Med. 2008;14:213–221. 18193059 10.1038/nm1684

[13] Pan J, Yan S, Gao JJ, et al. In-vivo organ engineering: perfusion of hepatocytes in a single liver lobe scaffold of living rats. Int J Biochem Cell Biol. 2016;80:124–131. 27720934 10.1016/j.biocel.2016.10.003

[14] Sullivan DC, Mirmalek-Sani SH, Deegan DB, et al. Decellularization methods of porcine kidneys for whole organ engineering using a high-throughput system. Biomaterials. 2012;33:7756–7764. 22841923 10.1016/j.biomaterials.2012.07.023

[15] Nahmias Y, Schwartz RE, Hu WS, et al. Endothelium-mediated hepatocyte recruitment in the establishment of liver-like tissue in vitro. Tissue Eng. 2006;12:1627–1638. 16846358 10.1089/ten.2006.12.1627

[16] Mirmalek-Sani SH, Sullivan DC, Zimmerman C, et al. Immunogenicity of decellularized porcine liver for bioengineered hepatic tissue. Am J Pathol. 2013;183:558–565. 23747949 10.1016/j.ajpath.2013.05.002 PMC3730770

[17] Orlando G, Farney AC, Iskandar SS, et al. Production and implantation of renal extracellular matrix scaffolds from porcine kidneys as a platform for renal bioengineering investigations. Ann Surg. 2012;256:363–370. 22691371 10.1097/SLA.0b013e31825a02ab

[18] Barakat O, Abbasi S, Rodriguez G, et al. Use of decellularized porcine liver for engineering humanized liver organ. J Surg Res. 2012;173:12. 10.1016/j.jss.2011.09.033 22099595

[19] Devalliere J, Chen Y, Dooley K, et al. Improving functional re- endothelialization of acellular liver scaffold using REDV cell-binding domain. Acta Biomater. 2018;78:151–164. 30071351 10.1016/j.actbio.2018.07.046 PMC6261340

[20] Shirakigawa N, Takei T, Ijima H. Base structure consisting of an endothelialized vascular-tree network and hepatocytes for whole liver engineering. J Biosci Bioeng. 2013;116:740–745. 23770123 10.1016/j.jbiosc.2013.05.020

[21] Crapo PM, Gilbert TW, Badylak SF. An overview of tissue and whole organ decellularization processes. Biomaterials. 2011;32:3233–3243. 21296410 10.1016/j.biomaterials.2011.01.057 PMC3084613

[22] Croce S, Peloso A, Zoro T, et al. A hepatic scaffold from decellularized liver tissue: food for thought. Biomolecules. 2019;9:813. 10.3390/biom9120813 PMC6995515 31810291

[23] Song JJ, Ott HC. Organengineeringbasedondecellularized matrix scaffolds. Trends Mol Med. 2011;17:424–432. 21514224 10.1016/j.molmed.2011.03.005

[24] Kim JJ, Hou L, Huang NF. Vascularization of three-dimensional engineered tissues for regenerative medicine applications. Acta Biomater. 2016;41:17–26. 27262741 10.1016/j.actbio.2016.06.001 PMC4969172

[25] Evans DW, Moran EC, Baptista PM, et al. Scale-dependent mechanical properties of native and decellularized liver tissue. Biomech Model Mechanobiol. 2013;12:569–580. 22890366 10.1007/s10237-012-0426-3

[26] Moran EC, Baptista PM, Evans DW, et al. Evaluation of parenchymal fluid pressure in native and decellularized liver tissue. Biomed Sci Instrum. 2012;48:303–309. 22846298

[27] Sánchez-Romero N, Sainz-Arnal P, Pla-Palacín I, et al. The role of extracellular matrix on liver stem cell fate: a dynamic relationship in health and disease. Differentiation. 2019;106:49–56. 30878881 10.1016/j.diff.2019.03.001

[28] Park KM, Park SM, Yang SR, et al. Preparation of immunogen-reduced and biocompatible extracellular matrices from porcine liver. J Biosci Bioeng. 2013;115:207–215. 23068617 10.1016/j.jbiosc.2012.08.023

[29] Hussein KH, Park KM, Teotia PK, et al. Sterilization using electrolyzed water highly retains the biological properties in tissue-engineered porcine liver scaffold. Int J Artif Organs. 2013;36:781–792. 24338653 10.5301/ijao.5000246

[30] Mattei G, Di Patria V, Tirella A, et al. Mechanostructure and composition of highly reproducible decellularized liver matrices. Acta Biomater. 2014;10:875–882. 24184179 10.1016/j.actbio.2013.10.023

[31] Hussein KH, Park KM, Kang KS, et al. Heparin-gelatin mixture improves vascular reconstruction efficiency and hepatic function in bioengineered liver. Acta Biomater. 2016;38:82–93. 27134015 10.1016/j.actbio.2016.04.042

[32] Wu Q, Li Y, Wang Y, et al. The effect of heparinized decellularized scaffolds on angiogenic capability. J Biomed Mater Res A. 2016;104(12):3021–3030. 27459086 10.1002/jbm.a.35843

[33] De Rossi G, Whiteford JR. Syndecans in angiogenesis and endothelial cell biology. Biochem Soc Trans. 2014;42(6):1643–1646. 25399583 10.1042/BST20140232

[34] Couchman JR. Transmembrane Signaling Proteoglycans. Annu Rev Cell Dev Biol. 2010;26:89–114. 20565253 10.1146/annurev-cellbio-100109-104126

[35] Baeyens N, Mulligan-Kehoe MJ, Corti F, et al. Syndecan 4 is required for endothelial alignment in flow and atheroprotective signaling. Proc Natl Acad Sci USA. 2014;111:17308–17313. 25404299 10.1073/pnas.1413725111 PMC4260558

[36] Bellin RM, Kubicek JD, Frigault MJ, et al. Defining the role of syndecan-4 in mechanotransduction using surface-modification approaches. Proc Natl Acad Sci USA. 2009;106:2102–22107. 10.1073/pnas.0902639106 PMC2796905 20080785

[37] Karimi F, Daniel E. Heath, biomaterials functionalized with nanoclusters of integrin- and syndecan-binding ligands improve cell adhesion and mechanosensing under shear flow conditions. J Biomed Mater Res A. 2020 June 3. DOI:10.1002/jbm.a.37024 32490581

[38] Hanahan D, Folkman J. Patterns and emerging mechanisms of the angiogenic switch during tumorigenesis. Cell. 1996;86(3):353–364. 8756718 10.1016/s0092-8674(00)80108-7

[39] Chen-L-L. SDC4 gene silencing favors human papillary thyroid carcinoma cell apoptosis and inhibits epithelial mesenchymal transition via Wnt/β-catenin pathway. Mol Cells. 2018 Sept 30;41(9):853–867. 30165731 10.14348/molcells.2018.0103 PMC6182223

[40] Ochieng J. Extracellular histones are the ligands for the uptake of exosomes and hydroxyapatite-nanoparticles by tumor cells via syndecan-4. FEBS Lett. 2018 Oct;592(19):3274–3285. 30179249 10.1002/1873-3468.13236 PMC6188801

[41] Habes C. Sulfated glycoaminoglycans and proteoglycan syndecan-4 are involved in membrane fixation of LL-37 and its pro-migratory effect in breast cancer cells. Biomolecules. 2019 Sept 12;9(9):481. 10.3390/biom9090481 PMC6769752 31547381

