# Supplementary material for: Syndecan-4 promotes vascular beds formation in tissue engineered liver via thrombospondin 1
Source: Bioengineered. 2020 Nov 29;11(1):1313–24. doi: 10.1080/21655979.2020.1846897 (PMC8291860; doi:10.1080/21655979.2020.1846897)
Supplement: Supplemental Material [file KBIE_A_1846897_SM6043.docx]

**
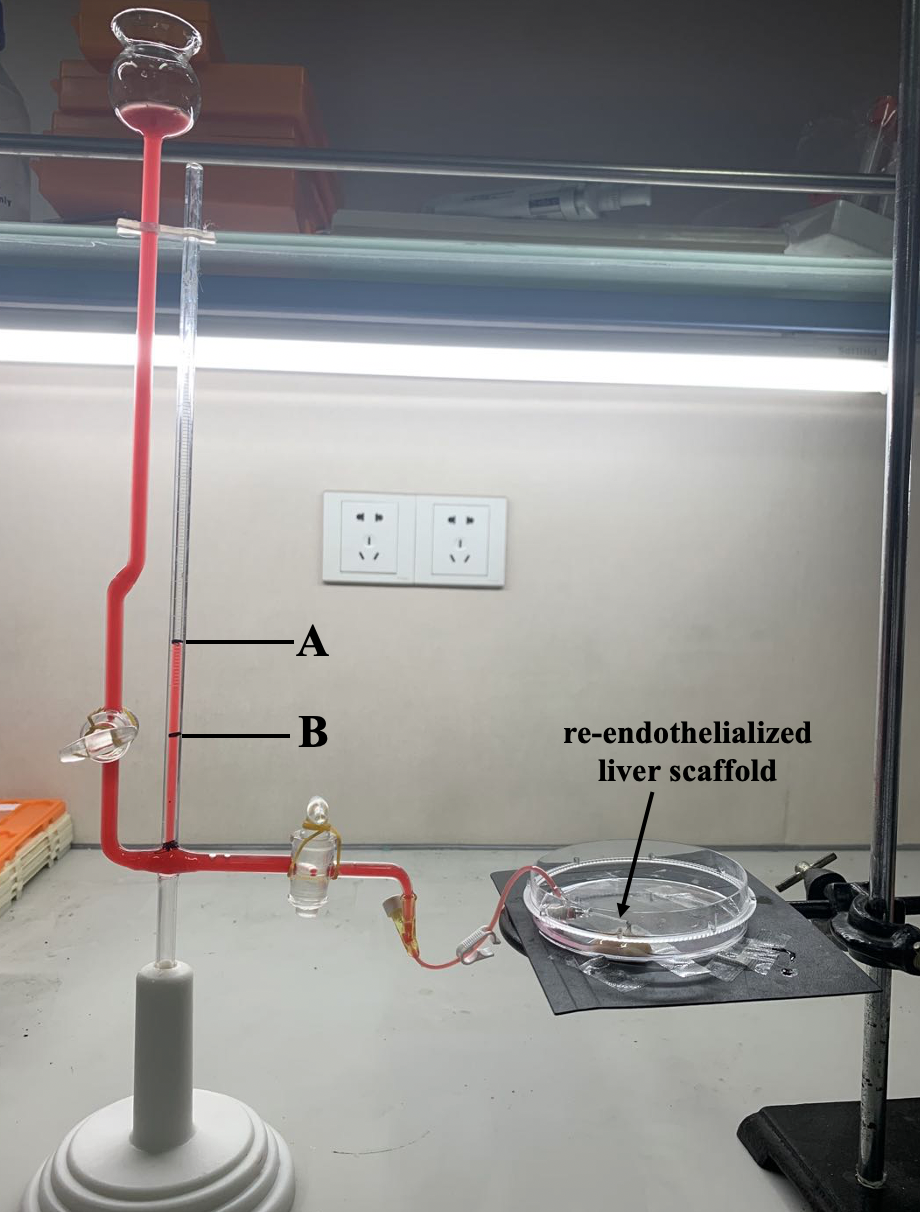
**

**Table. the flow rates in different repopulated liver**

|  | First time | Second time | Third time |
| --- | --- | --- | --- |
| isolated mouse liver  (not decellularization) | 1.9 ml/min | 1.8 ml/min | 1.8 ml/min |
| re-endothelialized liver scaffold with SDC4-NC EA.hy926 cells | 0.8 ml/min | 0.7 ml/min | 0.5 ml/min |
| re-endothelialized liver scaffold with SDC4 overexpressed EA.hy926 cells | 1.3 ml/min | 1.2 ml/min | 1.0 ml/min |

**Methods**

Since we do not have the experimental conditions such as radionuclide tracing, ultrasound or direct flow meter, we designed a simple and rough flow rate measurement device.

Device introduction:

The device was modified from a burette, and the burette on the right has a scale to read the volume (minimum scale is 0.01 mL). The pressure difference between point B and the bottom of the burette was equal to the inherent resistances of the entire pipeline and scaffold (measured by normal saline). The height difference between point A and point B is 4 cm, and the liquid level difference *V* is 0.27 mL. The burette is connected with scaffold through the indwelling needle, the bottom of the burette is at the same level with the scaffold, and there is no liquid in petri dish.

Introduction to fluid:

The fluid was the whole blood contributed by the first author (peripheral blood: normal saline=1:2, 0.2% heparin). The viscous resistance of the fluid is not considered. The density of the fluid is approximately 1g/mL.

Calculation:

According to the physiological data of the mouse liver (central venous pressure=5.9±2.0 cmH_2_0, portal pressure=8.09±2.47 cmH_2_O), without considering the flow of the hepatic artery, we believe that the pressure difference before and after the mouse liver is 2±2cm H_2_O, and this pressure difference presents a pulsed distribution. This is the reason why the height difference between A and B is 4 cm, which guarantees the average pressure difference is 2 cmH_2_O. According to Bernoulli's principle, the gravitational potential energy of the fluid with a height difference of 4 cm is completely converted into the kinetic energy. Therefore, as long as the time difference *T* of the liquid level falling from point A to point B is recorded, the average flow rate can be approximately calculated as *V/T*. And it is foreseeable that as the coagulation progresses, once the process of dropping the liquid level from A to B is repeated again and again, *T* would increase and *V/T* would decrease until the vascular bed of scaffold is completely blocked by the blood clot. We repeated this process three times for each scaffold model.

**Result**

Since we have excluded the inherent resistances of the entire pipeline and scaffold, V/T is only affected by the resistance generated during the coagulation process. The initial flow rate of the isolated mouse liver (not decellularization) was almost the same as reported [1-2], but as the coagulation progressed, the flow rate dropped significantly. The flow rate of the scaffold lined with the SDC4 overexpressed endothelial cells was significantly better than that of the SDC4-NC re-endothelialized liver scaffold.

1. Xie C, Wei W, Zhang T, et al. Monitoring of systemic and hepatic hemodynamic parameters in mice. J Vis Exp. (2014): e51955.

2. Albuszies G, Radermacher P, Vogt J, et al. Effect of increased cardiac output on hepatic and intestinal microcirculatory blood flow, oxygenation, and metabolism in hyperdynamic murine septic shock. Crit Care Med. (2005) 33: 2332-8.
